# Correlation between BRAF mutational status and clinical response to pembrolizumab in advanced melanoma patients

**DOI:** 10.1186/2051-1426-3-S2-P134

**Published:** 2015-11-04

**Authors:** Ester Simeone, Antonio Maria Grimaldi, Lucia Festino, Diana Giannarelli, Marco Palla, Corrado Caracò, Marcello Curvietto, Assunta Esposito, Miriam Paone, Giovanni Rinaldi, Maria Chiara Grimaldi, Nicola Mozzillo, Paolo A Ascierto

**Affiliations:** 1 O.U. Melanoma, Cancer Immunotherapy and Innovative Therapies Istituto Nazionale Tumori di Napoli Fondazione, Napoli, Italy; 2 Regina Elena National Cancer Institute, Rome, Roma, Italy; 3 O.U. Melanoma and Sarcoma Surgery Istituto Nazionale Tumori di Napoli Fondazione “G. Pascale”, Napoli, Italy; 4 Catholic University of Sacred Heart, Roma, Italy; 5 Istituto Nazionale Tumori Fondazione G. Pascale, Naples, Italy

## Background

About 50% of melanoma (MM) have BRAF V600 mutation and BRAF inhibitors, such as vemurafenib and dabrafenib, have shown an high response rate (RR) and overall survival (OS) improvement in BRAF-mutated MM patients (pts). Pembrolizumab, a monoclonal antibody anti-PD-1, has shown a significant improvement in RR and OS as well, in both MM pts naïve and previously treated with ipilimumab. As an immunotherapy, pembrolizumab should not be affected by BRAF mutational status and the aim of our analysis is to verify whether pembrolizumab is active regardless the BRAF mutational status .

## Methods

Inside the expanded access program (EAP), pembrolizumab was given in pts progressing after ipilimumab at dosage of 2 mg/kg every 3 weeks until PD or unacceptable toxicity. At our Institution 47 pts (25M, 22F) were treated. The median age was 49 years (range 28-70). All pts were stage M1c. 40 (85.1%) pts had cutaneous MM, 5 (10.6%) had ocular MM and 2 (4.3%) mucosal MM. 16 out of 47 (34%) had BRAF V600 mutation, and all mutated pts were previously treated with BRAF inhibitors as per protocol. In this retrospective analysis we evaluated the correlation between BRAF mutational status and response to pembrolizumab.

## Results

Data on RR and progression free survival (PFS) are available for all 47 pts enrolled in the EAP. From this analysis we excluded ocular MM because it's considered a distinct entity with a different biology. Among the 16/42 (38,1%) pts with BRAF mutation, RR was of 12.5% (2/16) compared with a RR of 36.4% (9/26) observed in BRAF wild-type cohort. The difference was not statistically significant (p 0.16). Disease control rate (DCR) in pts with BRAF mutation resulted 18.6% (3/16) compared with 65.4% DCR (17/26) of BRAF wild-type cohort. The difference was statistically significant (p 0.005). Median PFS of BRAF mutated pts was 3 months (range 2.3 – 3.7), while was not reached (2 – 8+) in BRAF wild-type cohort. The difference resulted statistically significant (p 0.001).

## Conclusions

Previous BRAF inhibitors treatment may affect response to pembrolizumab. Further studies are needed to verify this very preliminary observation.

